# Factors contributing to patient safety incidents in primary care: a descriptive analysis of patient safety incidents in a French study using CADYA (categorization of errors in primary care)

**DOI:** 10.1186/s12875-018-0803-9

**Published:** 2018-07-19

**Authors:** M. Chaneliere, D. Koehler, T. Morlan, J. Berra, C. Colin, I. Dupie, P. Michel

**Affiliations:** 1 Family Medicine Department, Université Claude Bernard Lyon 1, 8 avenue Rockefeller, 69008 LYON, France; 2 Hospices Civils de Lyon, 3 quai des Célestins, 69002 Lyon, France; 3 University of Lyon, Université Claude Bernard Lyon 1, Université Saint-Etienne, HESPER EA 7425 69008 LYON, F-42023 Saint-Etienne, France; 4 Société de Formation Thérapeutique du Généraliste, 233 Bis Rue de Tolbiac, 75013 Paris, France

**Keywords:** Patient safety, Primary care, Patient safety incident, Contributing factors, Human factor

## Abstract

**Background:**

Patient safety incidents (PSIs) frequently occur in primary care and are often considered to be preventable. Better knowledge of factors contributing to PSIs is required to build safer care. The aim of this work was to describe the underlying factors, specifically the human factors, that are associated with PSIs in primary care using CADYA (“CAtégorisation des DYsfonctionnements en Ambulatoire” or “Categorization of Errors in Primary Care”).

**Methods:**

We followed a mixed method with content analysis and coding in CADYA of PSIs reported in the ESPRIT study, a French cross-sectional survey of primary care. For each incident, a main contributing factor (MD) and, if applicable, a secondary contributing factor (SD) were identified. Several descriptive keywords from an incremental glossary have been suggested to describe each identified human factor (attitudes or behaviours). A descriptive statistical analysis was then conducted.

**Results:**

Among the 482 PSIs reported in the ESPRIT study, from 13,438 acts reported by 127 participating general practitioners (GPs), we identified 590 contributing factors (482 MDs and 178 SDs). Overall, 35% were related to the care process, 30% to human factors, 22% to the healthcare environment and 13% to technical factors. The contributing factors, in decreasing order of frequency, were communication errors (13.7%), human factors related to healthcare providers (12.9%) and human factors related to patients (12.9%). The human factors were mainly related to ‘lack of attention’, ‘stress’, ‘anger’ and ‘fatigue’.

**Conclusions:**

Our results tend to prove that human factors are often involved in PSIs in primary care, with GPs and patients being equally responsible. Beyond the identification of communication errors, often found in other international research, we have described the attitudes and behaviours contributing to unsafe care. Further research exploring the links between working conditions and human factors is required.

**Electronic supplementary material:**

The online version of this article (10.1186/s12875-018-0803-9) contains supplementary material, which is available to authorized users.

## Background

Patient safety incidents (PSIs) have been reported in primary care under various names [1]. Fifteen years ago, the report ‘To err is human’ led to an international awareness of the frequency and gravity of PSIs [2]. In 2007 [3], the frequency of PSIs in primary care was estimated to be from 2 to 240 incidents per 1000 encounters, and 45–76% were considered to be preventable. The wide range in PSI frequencies can be explained by the type of study (prospective or retrospective), the data collection methods, the multiple proposed definitions of PSIs and the trend of underreporting [3, 4]. In France, two prospective studies [5, 6] have estimated the frequency of PSIs in primary care to be between 0.5 and 1 event per day per general practitioner (GP). PSIs in the primary care setting significantly differ from those in hospitals [7–9] regarding their contributing factors [10]. To classify PSIs, several taxonomies, both specific and not specific to primary care, exist [11–18]. The World Health Organization (WHO) classification [18] is the most universal, but the consequent number of items that compose this taxonomy limits its current use. The first results of the French national survey on PSIs in primary care (ESPRIT) study [5] were based on the Threats to Australian Patient Safety (TAPS) version of the International Taxonomy of Medical Error in Primary Care [12] (ITME-PC) and the Tempos classification [14]. The TAPS Taxonomy [12] describes the nature of the incident according to 2 main types of errors: errors related to the care process with 5 sublevels and errors of knowledge or skills of the actors with 2 sublevels (diagnosis and patient management). The Tempos classification [14] uses time in classifying factors contributing to incidents according to five categories: the tempo of illness and treatment, the tempo of the physician, the tempo of the office, the tempo of the patient and the tempo of the health system. CADYA (Additional file 1: Appendix S1), which can be translated as Categorization of Errors in Primary Care, is a French taxonomy [17] that provides a complementary approach by describing more accurately the factors contributing to PSIs, especially the human factors, as suggested by research in aeronautics [19]. Four main levels are explored: technical factors, the environment (action background), processes related to the decision or care process (skills), and human factors (physical conditions, psycho-relational elements, attitudes or behaviours).

The main objective of the present study was to describe the incidents of the ESPRIT study [5] using the CADYA taxonomy to have better knowledge of their contributing factors.

## Methods

A mixed method approach, combining qualitative analysis (content analysis) and quantitative analysis (descriptive analysis), has been used. Because the work is based on the results of the ESPRIT study [5], we only present the most relevant methodological elements.

### ESPRIT study’s methodological aspects

The ESPRIT study [5] was a cross-sectional study. Its aim was to describe the incidence and nature of PSIs in primary care general practice settings. The incidence was defined as the ratio of the number of medical encounters (or patient contacts) with one PSI and the total number of medical encounters included during the study period.

The target population was all of the patients seen or contacted by GPs working in France. The source population in the ESPRIT study [5] was composed of all of the patients seen or contacted by a GP from the GROG (Groupes régionaux d’observation de la grippe). The GROG was a nationwide network for influenza surveillance in France that included more than 800 volunteer GPs. The GROG was considered sufficiently representative of the French GPs to support several epidemiological studies in general practice in France and Europe. The sample was composed of GPs that were randomly selected from the GROG physicians. A minimum sample size of 120 GPs, with a gender distribution consistent with the national distribution (70 men and 50 women), was achieved. The GPs were invited to participate in the study by telephone. The GROG coordination team trained all participating GPs on the study protocol and on the data collection method by phone and by a written procedure sent by mail. A specific training on PSIs in primary care was also provided by phone or by web with a video (available on the study website). The aim was to ensure that all GPs had a same understanding of what constituted an incident to be reported in an effort to reduce the selection bias and reporting bias.

### Data collection method

Data collection for each GP occurred for one week, which was chosen by the GP within the six-week data collection period from May 2013–June 2013. The definition of an incident was proposed by focus groups and approved by consensus among primary care experts and GP representatives. An incident was defined as “an event or circumstance that could have resulted, or did result, in harm to a patient, and which should not be repeated again” [20]. An “act” was defined as any patient contact, including a consultation, visit, or intervention, in a nursing home or by telephone**.** Participating GPs completed a daily PSI reporting webform throughout the data collection period. That form was developed by the ESPRIT study expert group after a literature review. Three forms were used: a GP profile form, a register of acts (all acts during the collection week) and a PSI reporting form containing 25 questions capturing a description of each PSI along with contributing factors and consequences. The data that were collected were compiled on a secure website; data quality was reviewed by an expert group (eight GPs competent in the patient safety field and two epidemiologists). At the end of the data collection process, each reported event was reviewed during an expert group seminar to verify the situation as a PSI. The data quality control process was thus carried out in several stages.

### Ethical aspects

No nominative, sensitive or personal health data concerning patients were systematically collected. The ESPRIT study [5] was therefore not, strictly speaking, in the field of biomedical research (fields of application of the French reference methodology MR 001), nor was it within the framework of Chapter X of the French Data Protection Act. For this practice-oriented study, the only nominative data collected were GP profile data that were available in a database declared to the French CNIL (number 1684220). GPs provided their written informed consent to participate by email. The National Council of the French Medical Association was also informed due to the indemnification of the participating GP. All ethical approvals of the study were given by the Ile-de-France Paris III committee and by the CNIL (French Data Protection Authority).

### Work methodological aspects

#### Recruiting coders

Two GPs from the Family Medicine Department of the Claude Bernard University Lyon 1 (DK and TM) volunteered to analyse the ESPRIT study PSIs. An expert GP (MC) who was involved in patient safety in primary care trained the two recruiting coders to reach a shared understanding of, and a similar level of expertise on, the root cause analysis and the use of CADYA.

### Coding process

All of the PSIs related to a patient were extracted from the ESPRIT study database. For each PSI, the investigators were required to independently search for the underlying factors contributing to their occurrence according to a qualitative approach (content analysis). Then, the investigators had to select a main contributing factor (MD), called “dysfunction” in French, and, if relevant, a secondary contributing factor (SD) in CADYA [17]. The choice between designating the factor as a MD or SD depended on the estimated impact of the contributing factor to the PSI. In the case of a factor related to a human attitude or behaviour, the investigators had to specify a keyword to describe its nature (e.g., “anger”, “stress” or “tiredness”) according to an incremental glossary (a keyword that was already identified, or, if necessary, a new keyword). The investigators had to describe the human factor with as much accuracy as possible. They regularly compared their coding after every 50 reports. In cases of disagreement, the investigators were required to reach a consensus. At the end of data extraction, all coding was approved by the expert GP (MC). The review of each investigator, the consensus and the expert final review were compiled into a single Excel® file. Finally, the investigators checked, for a second time, that there were no redundant keywords in the thesaurus. If necessary, they gathered redundant keywords into a single consensual keyword to obtain a reduced, but more precise, thesaurus.

### Statistical analysis

Descriptive data analysis was conducted according to the dimensions of CADYA. The frequencies of agreements between the two coders and between coders and experts were estimated. The inter-rater reliability was analysed with a kappa coefficient [21], which was expressed as follows: (% observed agreement - % agreement due to chance) / (1 -% agreement due to chance). The interpretation of the calculated kappa coefficients followed the six classes by Landis and Koch [22].

## Results

### Main ESPRIT study results [5]

A total of 13,438 acts were recorded by the 127 participating GPs; 82% (*n* = 11,023) were office consultations, 8% (*n* = 1090) were telephone contacts, 8% (*n* = 1022) were home visits and 2% (*n* = 303) were visits at nursing homes. The GPs in the sample were older and worked more often in groups and more often in rural areas than the population of GPs in France (Table 1**)**. The average number of patient encounters per day was 21. The average age of the patients was 48 years, and 4013 patients (30%) had a chronic or long-term condition.

**Table 1:** Description of the participant GPs

Variables	Answers	French National Data	ESPRIT study data (%)	*p*-value
Gender	Men Women	66% 34%	60% 40%	NS
Average act per week		102	100	NS
Average age		51 years	54 years	< 0.05
Mode of practice	Solo Group practice	52% 48%	40% 60%	< 0,05
Area of practice	Rural Urban	15% 85%	17% 83%	< 0,05

A total of 694 events were reported by the participating GPs, 674 (97%) of which were reported as being related to a patient and 20 (3%) of which were reported as being not related to a particular patient. Additionally, of these 694 PSIs, 212 (31%) were not PSIs as defined. Thus, 482 (69%) PSIs were validated by experts, 475 (99%) of which were related to one patient and 7 (1%) of which were non-patient related (usually computer glitches affecting a whole range of medical encounters). Of these 475 PSIs, 52% occurred in the office, 37% occurred in patients’ homes and 12% occurred in nursing homes. Additionally, 55% of the patients were women. The average age of the patients was 56 years (standard deviation: 25 years), with a range from zero years (four patients) to 100 years (one patient). Nearly half of the patients (40%) were retirees.

There were no clinical consequences for 73% of PSIs, and for 25%, there was a temporary disability. Only 2% of the PSIs were serious in the ESPRIT study (*n* = 9), being associated with a life-threatening or permanent disability (i.e., 0.07% of the 13,438 patient encounters observed in the study).

### General data

The investigators reviewed the 482 validated PSI reports. They excluded 74 incidents due to unpredictable adverse drug reactions (no identifiable contributing factors). Therefore, 408 reports were ultimately included for review. The investigators identified 408 MDs and 182 SDs (ratio of 0.45) for a total of 590 factors (TDs).

### Frequency and nature of contributing factors

Table 2 shows the distributions of the MDs, SDs and TDs according to the CADYA items.

**Table 2:** Distributions of the main (MD), secondary (SD) and total number of (TD) contributing factors

Dimensions	Main contributing factor *n* (% of MD)	Secondary contributing factor *n* (% of SD)	Total contributing factor *n* (% of TD)
ENVIRONMENTAL FACTORS	93 (22.8)	37 (20.3)	130 (22)
PATIENT’S SOCIAL CONTEXT	5 (1.2)	5 (2.7)	10 (1.7)
BACKGROUND OF CARE	38 (9.3)	20 (11)	58 (9.8)
Unplanned consultation	10 (2.4)	0 (0)	10 (1.7)
Place of care	6 (1.5)	8 (4.4)	14 (2.4)
Workload management	22 (5.4)	12 (6.6)	34 (5.7)
DISRUPTIVE ELEMENT	27 (6.6)	7 (3.9)	34 (5.7)
HEALTH SYSTEM	23 (5.7)	5 (2.7)	28 (4.7)
Healthcare service	20 (5)	2 (1)	22 (3.7)
Financial or administrative issue	3 (0.7)	3 (1.7)	6 (1)
HUMAN FACTORS	89 (21.8)	86 (47.3)	175 (29.7)
LINKED TO THE PATIENT	45 (11)	31 (17)	76 (12.9)
LINKED TO THE PROVIDER	35 (8.6)	41 (22.5)	76 (12.9)
LINKED TO OTHER PROVIDERS	4 (1)	7 (3.9)	11 (1.9)
LINKED TO A THIRD PARTY	5 (1.2)	7 (3.9)	12 (2)
TECHNICAL FACTORS	67 (16.4)	9 (4.9)	76 (12.9)
EQUIPMENT	21(5.2)	2 (1)	23 (3.9)
Failure, malfunction, unavailability	19 (4.7)	1 (0.5)	20 (3.4)
Incorrect use	2 (0.5)	1 (0.5)	3 (0.5)
INFORMATION SYSTEM	46 (11.3)	7 (3.9)	53 (8.9)
Incorrect or missing data	34 (8.3)	4 (2.2)	38 (6.4)
Failure of the communication system	12 (3)	3 (1.7)	15 (2.5)
PROCESS OF CARE	159 (39)	50 (27.5)	209 (35.4)
COGNITIVE DIMENSION	56 (13.8)	9 (5)	65 (11)
Lack in initial training	26 (6.4)	3 (1.7)	29 (4.9)
Incorrect recall (after training)	13 (3.2)	2 (1)	15 (2.5)
Incorrect synthesis	17 (4.2)	4 (2.2)	21 (3.6)
CARE PROCEDURE	34 (8.3)	14 (7.7)	48 (8.1)
Inappropriate or unachieved procedure	31 (7.6)	13 (7.1)	44 (7.5)
Lack of protocol	3 (0.7)	1 (0.6)	4 (0.6)
CARE COORDINATION	69 (16.9)	27 (14.8)	96 (16.3)
Communication failure	59 (14.5)	22 (12.1)	81 (13.7)
Lack of (or incorrect) monitoring	9 (2.2)	5 (2.7)	14 (2.4)
Lack of response after feedback	1 (0.2)	0 (0)	1 (0.2)
TOTAL	408 (100)	182 (100)	590 (100)

Table 3 shows some examples of the factors identified by the dimensions and sub-dimensions of CADYA.

**Table 3:** Examples of contributing factors by the dimensions and sub-dimensions of CADYA

Item	Examples of patient safety incidents (in the ESPRIT study)
ENVIRONMENTAL FACTORS	
Patient’s social context	Elderly, suffering from dementia, unsuitable assistance plan
Background of care	
Unplanned consultation	A woman took an appointment just for herself and came with her son
Place of care	Incomplete medical examination because the patient was seen at home
Workload management	Workload increased by adding too many consultations
Disruptive element	Phone call caused the physician to dismiss his patient
Health system	
Healthcare service	A medical specialist was required but unavailable on weekends
Financial or administrative issue	No general practitioner declared to social security
HUMAN FACTORS	
Linked to the patient	Aggressive patient (who felt rejected by her physician)
Linked to the provider	Stressed physician (bad news needed to be announced)
Linked to other providers	Pharmacist distracted when dispensing treatment
Linked to a third party	Indiscretion of the mother of a patient regarding her daughter
TECHNICAL FACTORS	
Equipment	
Failure, malfunction, unavailability	Failure of the computer server
Incorrect use	Wound following the inappropriate use of pedicure equipment
Information system	
Incorrect or missing data	Lack of discharge letter after hospitalization of a patient
Failure of the communication system	Ultrasound result was unreadable over the internet
PROCESS OF CARE	
Cognitive dimension	
Lack of initial training	Ignorance of drug contraindication
Incorrect recall (after training)	Insufficient exploration of thromboembolic risk
Wrong synthesis	Minimization of a chronic kidney disease
Care procedure	
Inappropriate or unachieved procedure	Coronary patient who stopped the statin on her own
Lack of protocol	Medical appointment for an emergency assigned too late by the secretary
Care coordination	
Communication failure	The nurse did not call the physician despite an unusual dosage
Lack of (or incorrect) monitoring	Lack of specialized ophthalmic monitoring despite serious uveitis
Lack of response after feedback	Diabetes mellitus non-equilibrated, without medical appointment, for several months despite several blood tests

**Fig. 1:**
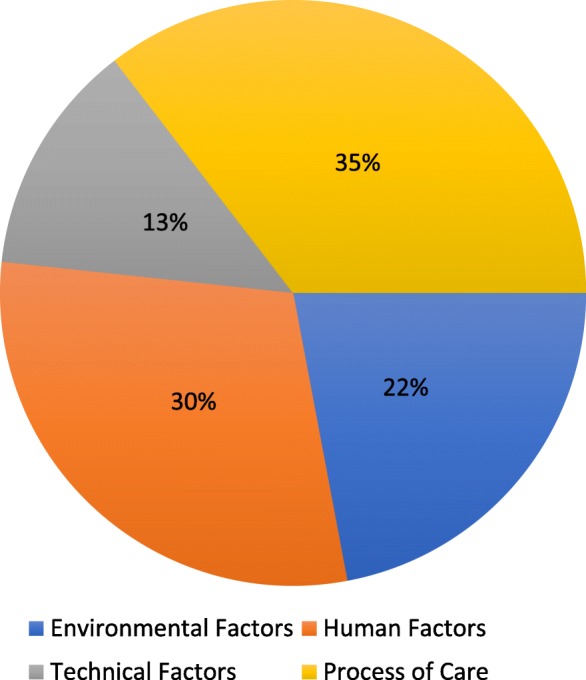
Distribution (%) of the total contributing factors by the main dimensions of CADYA

**Fig. 2:**
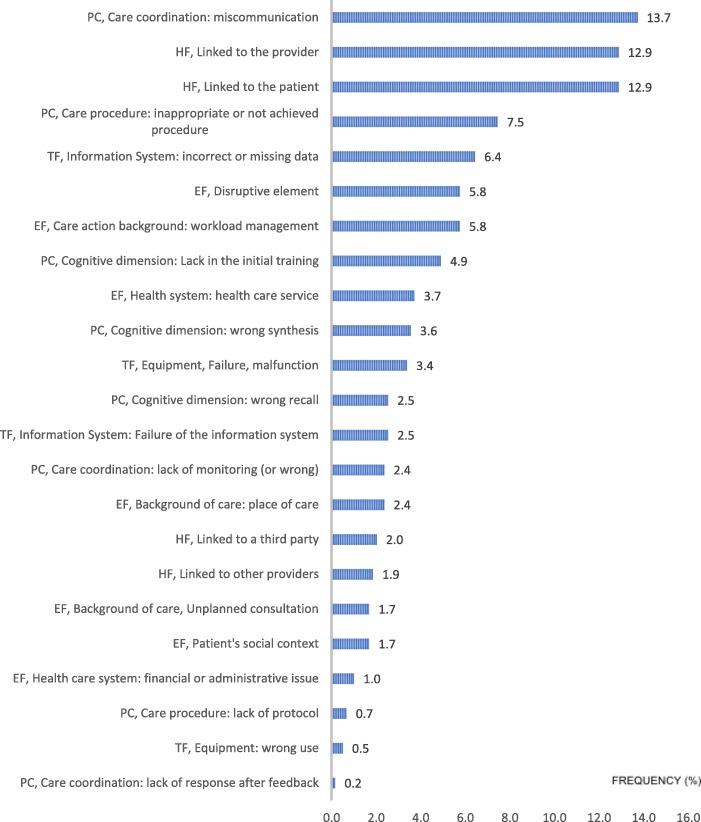
Distribution of all the identified contributing factors in CADYA (% of the total number). Legend: PC: Process of Care; HF: Human Factors; TF: Technical Factors; EF: Environmental Factors

Figures 1 and 2 show the distributions of the 590 contributing factors according to the main dimensions and sub-dimensions of CADYA, ranked in order of frequency.

### Analysis of main contributing factors (Fig. 3)

The MDs were related primarily to the care process (39%), communication errors (14.5%) and cognitive aspects (13.8, 6.4% of which were due to training defects) and secondarily to environmental factors (22.8%) and human factors (21.8%). Regarding the environmental factors, the management of workload (5.4%) and the occurrence of a disruptive element (6.6%), such as a phone call during the consultation, were responsible for more than half of these MDs. Human factors were more often related to the patient (11%) than to the health professionals (8.6%). When these factors were due to a technical factor (16.4%), it was mainly related to issues in the information system (8.3% due to missing or incorrect data in the medical chart).

**Fig. 3:**
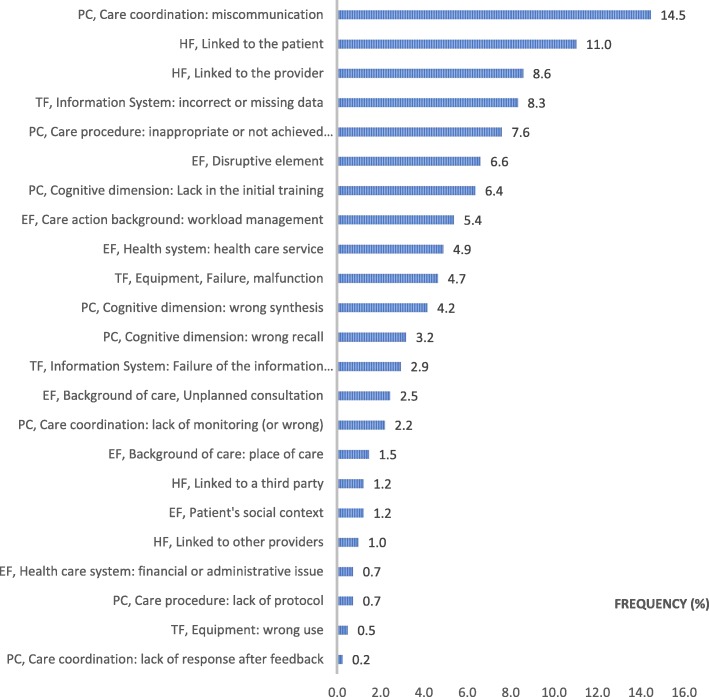
Distribution of the main contributing factors in CADYA (% of the total number). Legend: PC: Process of Care; HF: Human Factors; TF: Technical Factors; EF: Environmental Factors

### Secondary contributing factors (Fig. 4)

Almost half of the SDs were related to human factors (47.3%) and were linked more frequently to the GP (22.5%) than to the patient (17%). More than one quarter (27.5%) of the errors in the care process were identified as being from SDs, including communication errors (15%). Technical factors were still relatively low (4.9%), while those related to the environment (20.3%), including workload (6.6%) and place of care (4.4%), remained stable.

**Fig. 4:**
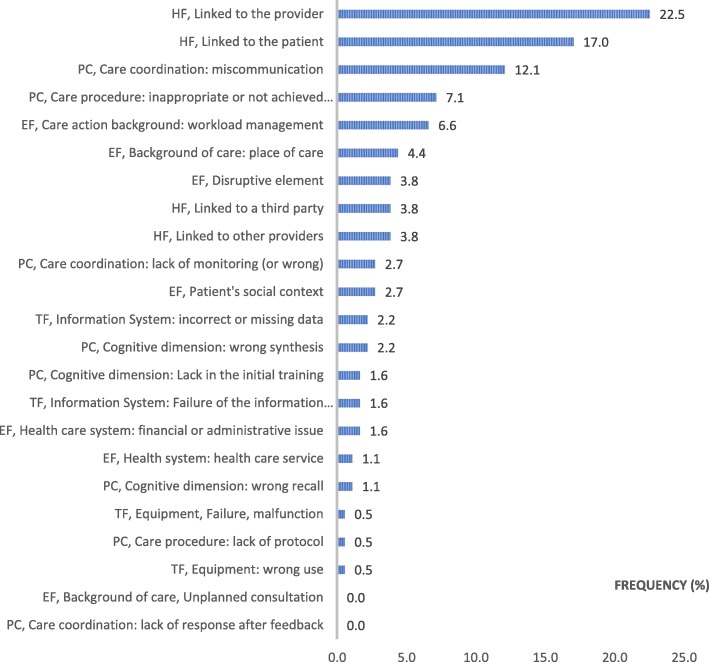
Distribution of the secondary contributing factors in CADYA (% of the total number). Legend: PC: Process of Care; HF: Human Factors; TF: Technical Factors; EF: Environmental Factors

### Nature of human factors

**Fig. 5:**
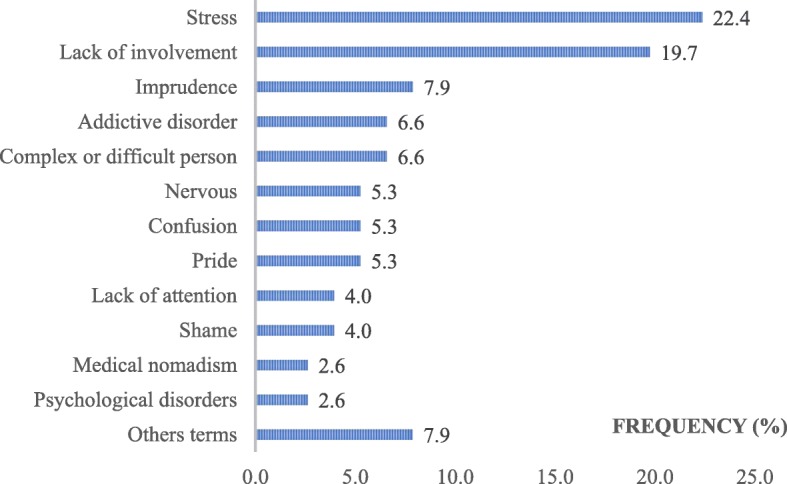
Keywords used to describe the human factors linked with the patient, as ranked by the frequency of use (%)

**Fig. 6:**
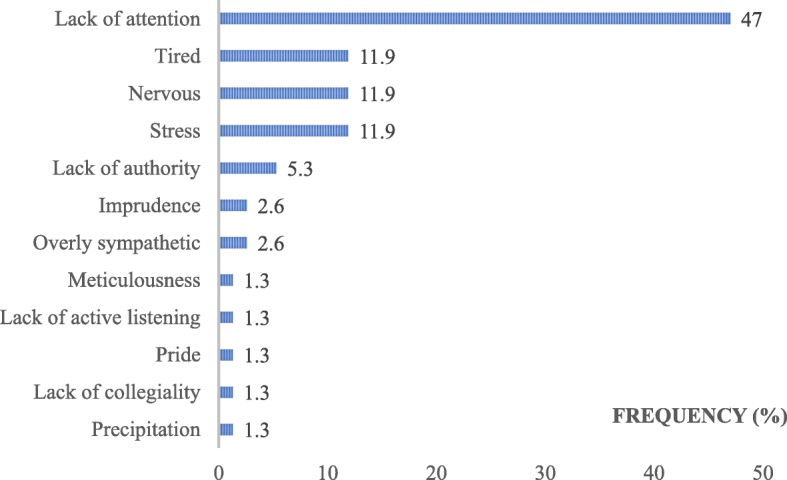
Keywords used to describe the human factors linked with the GP, as ranked by the frequency of use (%)

Overall, we identified 175 factors related to human attitudes or behaviours, divided similarly between MDs (*n* = 89) and SDs (*n* = 86), consisting of more than a quarter of all of the TDs (29.7%). These factors represented more than one-fifth of all MDs (21.8%) and almost half of all SDs (47.3%). In total, human factors related to either the GP or the patient constituted 87% of all the identified human factors (13% due to a third party and other caregivers). Figures 5 and 6 display the frequencies of keywords used to describe the human factors that are linked to the patient and to the GP, respectively. Table 4 shows a glossary of 15 keywords resulting from semantic harmonization by the investigators. Overall, 5 keywords represented 2/3 of all the human factors: ‘lack of attention’, ‘stress’, ‘lack of involvement’, ‘nervousness’ and ‘tiredness’.

**Table 4:** Keywords related to the human factor descriptions (glossary)

KEYWORDS AFTER SEMANTIC WORK	KEYWORDS IN THE STUDY	GLOBAL FREQUENCY OF USE IN GLOSSARY (% of all tags)
LACK OF ATTENTION	Lack of attention, Lapse in concentration, Absent-mindedness	27.5
STRESS	Anxiety, Stress, Anguish, Fear	15.5
LACK OF INVOLVEMENT	Lack of medical observance, Non-involvement, Refusing care, Lag (to consult), Lack of active listening	10.3
NERVOUSNESS	Anger, Irritation, Violence, Aggressiveness	8
TIREDNESS	Tiredness, Asthenia	5.7
IMPRUDENCE	Negligence, Clumsiness	5.1
COMPLEX OR DIFFICULT PERSON	Grouchy, Child-King, Claims	3.4
ADDICTIVE DISORDER	Addictive disorder	2.9
PRIDE	Self-love, Pride, Overconfidence, Vanity	2.9
LACK OF AUTHORITY	Lack of authority, Lack of bravery	2.2
MISUNDERSTANDING	Confusion, Misunderstanding	2.2
SHAME	Shame, Inconvenience, Shyness	1.7
PSYCHOLOGICAL DISORDERS	Psychological disorders	1.1
OVERLY SYMPATHETIC	Sympathetic, Overly empathic	1.1
IMPATIENCE	Impatience, Precipitation	1.1
OTHER WORDS	Falsehood, Medical nomadism, Impairment, Indiscretion, Meticulousness, Verbal comprehension issue, Personal emergency, Recklessness, Abusive request, Lack of collegiality	9.3
	TOTAL (%)	100

### Reproducibility analysis

The levels of agreement between the investigators were 64.5% for the main dysfunctions (311 of 482) and 67.4% for the secondary dysfunctions (325 of 482). The kappa coefficient for agreement between the investigators at the first level of CADYA (main dimensions) was estimated to be 0.685, suggesting a strong agreement. The statistical analysis was completed with the calculation of the kappa coefficient for the agreement between the investigators and the expert. The result was 0.028, which corresponds to a very low agreement.

## Discussion

### Key results

If we consider the overall contributing factors, our results suggest that errors in care processes are frequent. As highlighted in other research, communication errors are indeed of primary importance. More remarkably, our analysis has identified several attitudes and behaviours that contribute to unsafe care. Those attitudes and behaviours regard both professionals and patients and could often be described with only a 15-word glossary.

### Process of care

Among the contributing factors related to the process of care, miscommunication between healthcare professionals constitutes a main source of errors, as suggested by several international studies [11, 23–25]. This result suggests the need to develop communication training for health professionals (students or graduates). ‘Good communication skills’ is one of the seven pillars of a safety culture according to Sammer et al. [26]. For that reason, we think that it may be useful to share some training programmes among all students across all health disciplines. The students could then better understand the mutual roles of all primary care professionals. Other actions should be implemented and evaluated, e.g., promoting learning via games and simulations during medical studies to develop relationship skills in healthcare. To train primary care professionals to better structure their communication (especially oral), some standardized tools such as ‘SBAR’ (‘Situation-Background–Assessment-Recommendation’) [27], which is already used in some hospitals, could be deployed. Patients should also play a leading role in their safety. They should have access to similar tools to better communicate with professionals [28]. If these standardized communication tools were implemented in primary care, such tools could reduce the number of PSIs [29].

Contributing factors related to cognitive dimensions have been more frequently identified as being MDs rather than SDs. This difference seems to be related to the importance of the cognitive processes themselves. Indeed, even a small error at this level can produce a diagnostic or therapeutic issue, sometimes serious [30]. This issue leads to more frequent identification as a MD. In the ESPRIT study [5], 17% of the PSIs were related to ‘knowledge and skill errors’ according to the taxonomy of Makeham [12] and 30% to a ‘lack of knowledge or skills’ with the Tempos method [14]. Our analysis using CADYA is consistent with these results. As a consequence, to improve the quality and safety of primary care, it seems necessary to strongly promote the continuing education of all health professionals.

Missing or incorrect protocols constituted a marginal source of error in our research. However, GPs in the ESPRIT study [5] and, more broadly, French GPs do not write protocols in their daily practices. To explain the flaws in procedure executions, we must consider the independence in primary care of patients and professionals. The patient may choose to not take or insufficiently take the prescribed treatment, as shown by several studies [31], or they may defer an examination or new appointment. A health care professional may not always follow all of the guidelines, even if he or she knows that they exist. However, it is not necessarily a question of resistance to change [31, 32] but may be an evidence-based medicine approach [33]. Thus, decisions are also made in relation to the patient’s expectations and wishes, as well as to the caregiver’s context of care and representations.

### Human factors

Unsafe attitudes and behaviours have frequently been found and may be due to the particular patient-provider relationship in primary care. In French healthcare, as in many countries, the patient chooses professionals with more freedom in the primary care setting than in the hospital. CADYA distinguishes cognitive processes from other types of human factors. Other tools, such as the ALARM method (Association of Litigation And Risk Management) [34], have included these human factors in cognitive or decision process levels. The literature does not provide a definitive answer as to which is the best option. CADYA has been designed for educational purposes, e.g., university trainings, where the identification of unsafe attitudes and behaviours of healthcare workers is important for the development of a proper safety culture.

Overall, analysis of the glossary of terms used to describe the nature of human factors (GP and patient) showed three main types of factors: first, those related to a physical or mental impairment (e.g., ‘stress’, ‘extreme tiredness’, ‘worry’, or ‘distraction’); second, those related to a mental disorder (e.g., ‘aggressiveness’, ‘extreme nervousness’, or ‘addictive disease’); and finally, attitudes of relatively conscious disengagement of individuals during care (‘negligence’, ‘lack of commitment’, or ‘lack of compliance and absence of authority’).

It is tempting to establish a link between some human factors, especially those designating impaired performances, with unfavourable conditions of care. The ‘stress’ of GP and the ‘nervousness’ and ‘aggressiveness’ of some patients were sometimes secondary to a significant delay in the schedule of the appointments. Currently, providers in primary care are often confronted with multiple, sometimes paradoxical, injunctions, e.g., ‘to provide fewer appointments but with sufficient time to allow for patient-centred care’ but also ‘to open appointment access to have more consultations so that more patients can consult’. Therefore, human factors likely contribute to other factors, especially those related to cognitive processes.

### Environmental factors: Importance of favourable care conditions

Two elements are quite significant: an inadequate management of the workload and the existence of a “parasitic” or “disruptive element”. There is a wide variety of disruptive elements, including the interruption of the consultation by a third party in the office or by a phone call. The effect of this disruptive element is twofold. On the one hand, this disruption causes a break during the consultation, which can potentially alter the care provider’s reasoning process (blackout). On the other hand, it can induce “human impairment”, e.g., during the consultation, the GP answers the phone and learns of the death of a patient; this can induce a considerable emotional charge that is stressful for the healthcare provider. It seems logical, then, to limit the risk for disturbance during care, but there is a paradox: if they are unreachable or less easily reachable, the providers are less able to manage emergencies. In the ESPRIT study [5], unexpected emergencies were seldom identified because they were probably responsible for problems in the workload. This situation is consistent with the limited number of “true” emergencies in primary care [35] in modern health systems, where these acute events are ideally supported by dedicated services. To our knowledge, no study has specifically quantified the impact of an improvement in the environment of care on safety. However, this seems pragmatic, as it is known that anything that impairs communication within a team is likely to generate PSIs [36].

### Technical factors: First, gaps in information systems

GPs perform a small number of technical acts, which is consistent with a rather low frequency of ‘purely technical’ errors. Moreover, with the development of communication technologies in the past twenty years, many doubt that this type of error is experiencing a substantial decrease. The widespread use of secured software (including prescription-assistance modules) and electronic patient records help to limit some errors (e.g., illegible handwritten prescriptions), but this has also contributed to other issues, e.g., common ‘click’ errors in medication lists.

### CADYA

CADYA is complementary to the taxonomy of Makeham [12] and to the Tempos method [14], as it specifically explores processes related to medical decision-making and the unsafe attitudes and behaviours of actors. Regarding the main dimensions, the strong coefficient kappa between investigators argues for the robustness of CADYA. The poor kappa between the investigators and the expert seems paradoxical. This result is explained by the paradox of Feinstein and Ciccheti [37]: with a similar level of observed agreement, the estimated kappa is lower when the symmetrical balance is perfect. Despite a very high level of agreement, we obtained an abnormally low kappa coefficient.

CADYA is used in France by several morbidity and mortality review groups [17] and was supported by the French High Health Authority. It was also used during specific trainings on patient safety at Claude Bernard University Lyon 1, especially in those on Family Practice.

In its first version, CADYA did not provide a glossary to describe human factors related to attitudes and behaviours, and users could use any descriptive terms they wished. However, this flexibility created a limitation for a more standardized use. In addition, some users regretted the absence of a glossary that would facilitate the identification of human factors. The Glossary of human factors constitutes a useful upgrade in the most recent version of CADYA. This version is currently being used as part of the ongoing PRisM national study, which is dedicated to the assessment of a multifaceted programme on risk management in primary care. A full and validated English adaptation of CADYA is in progress, following the recommendations of the WHO [38].

### Strengths and limitations

Some limitations are inherent to the ESPRIT study [5]. The GPs were randomly selected within a network that was potentially biased towards reporting. According to us, this strategy was preferable to improve the number of PSIs reported by GPs and to have a better data collection quality. Yet, there is no indication that physicians enrolled in GROG are more concerned by issues related to patient safety. For that reason, we think that the findings - based on over 12,000 medical encounters - were not necessarily affected by this choice and the generalizability still remains acceptable. More than 200 events of the 694 incidents reported by GPs were deemed to not be PSIs by the expert group. This finding suggests that the level of understanding of the definition of PSI by participant GPs was not high, despite the time spent in GP training. This issue has been already described in previous studies [3, 4]. The main limitation of the ESPRIT study is that information on the patient health statuses remains limited. However, the literature on PSIs in primary care has already shown that factors such as an older patient age and an increased number of comorbidities have been associated with an increased risk for adverse events.

Regarding our work, the coding of the PSIs was performed by only 2 investigators. However, they have been previously trained to use the CADYA grid, and they regularly compared their coding results. The lack of information in some clinical descriptions, as well as the absence of time constraints to code the PSIs, may have also constituted a subjective bias in coding. For that reason, an expert validated the consistency of all the codes. The number of contributing factors identified was consistent with the limited information reported by the GPs in the ESPRIT study [5]. Thus, the reported events have often been reduced to one sentence or a few words. The investigators were instructed to only consider information without interpreting it to limit the risk of bias. Finally, the glossary consists of only 15 keywords; our experience is that attitudes and behaviours can, most of the time, be described with these keywords.

## Conclusions

PSIs are common in primary care, and research is needed to better understand them, as they differ from those in hospitals. We conducted an original study on a French national cohort of several hundred PSIs identified by GPs to describe their contributing factors. Our work confirms that, while there is a wide variety of contributing factors, the factors related to the process of care (in particular miscommunication) and the human factors related to unsafe attitudes and behaviours (e.g., stress, tiredness, etc.) represent major sources of PSIs.

We propose a 15-keyword glossary to describe these human factors. This glossary is applicable to all the actors, patients and caregivers alike engaged in care and can be useful in the initial training of medical students and in the continuing education of health providers. It remains, however, necessary to explore the links between these human factors and the working conditions of healthcare providers (e.g., the influence of the number of daily appointments and the influence of access without an appointment for patients), the patient’s life context and the healthcare system to improve patient safety in primary care.

## Additional file

Additional file 1: **Appendix S1.** Main dimensions and sub-levels of CADYA (Categorization of Errors in Primary Care). (DOCX 20 kb)

## Abbreviations

ALARM: Association of Litigation And Risk Management
CADYA: CAtégorisation des DYsfonctionnements en Ambulatoire (or Categorization of Errors in Primary Care)
CNIL: Commission Nationale de L’informatique et des Libertés
GP: General practitioner
GROG: Groupes régionaux d’observation de la grippe
ITME-PC: International Taxonomy of Medical Error in Primary Care
MD: Main contributing factor
PSI: Patient safety incident
SD: Secondary contributing factor
TAPS: Threats to Australian Patient Safety
WHO: World Health Organization
